# Prevalence and factors associated with post-traumatic stress disorder among internally displaced people in camps at Debre Berhan, Amhara Region, Ethiopia: a cross-sectional study

**DOI:** 10.1186/s12888-023-04570-w

**Published:** 2023-01-31

**Authors:** Belay Makango, Zewdie Aderaw Alemu, Tesfaye Solomon, Nigussie Lemma, Tewodros Girma, Tijani Mohammednur, Mikias Alayu, Yaregal Fufa

**Affiliations:** 1 Ethiopian Public Health Institute, P.O.Box: 1242, Addis Ababa, Ethiopia; 2 Department of Public Health, GAMBY Medical and Business College, Addis Ababa, Ethiopia; 3 Department of Public Health, College of Health Sciences, Debre Markos University, Debre Markos, Ethiopia

**Keywords:** Internally displaced people, Post-traumatic stress disorder, Ethiopia

## Abstract

**Background:**

Post-traumatic stress disorder (PTSD) is a common mental disorder after traumatic exposure that can have long-lasting physical and mental health consequences. In 2021, Ethiopia saw the highest number of internally displaced people (IDP) due to conflict and war with the scope of the internal displacement being very high in the study area and less attention has been given to mental health.

**Objective:**

To determine the prevalence and associated factors of PTSD among internally displaced people in camps at Debre Berhan, Ethiopia.

**Methods:**

A cross-sectional study was conducted from December 1–30, 2021 among 406 IDPs, who were selected by random systematic sampling from the registration and proportionally allocated to three IDP camps in Debre Berhan. Post-traumatic stress disorder was measured by the PTSD checklist (DSM-5). Data were collected through an interviewer-administered pre-tested questionnaire, entered into EpiData version 3.1, and analyzed by Statistical Package for Social Sciences version 25. Bivariate binary logistic regression was used to select candidate variables with *p* < 0.25. Multicollinearity was checked by using the variance inflation factor and it was less than 10. Model adequacy was checked by Hosmer & Lemeshow goodness of test (*p* > 0.05). In the multivariable binary logistic regression, the association between outcome and independent variables was declared at *p* < 0.05 with its adjusted odds ratio (AOR) at a 95% confidence level.

**Results:**

The prevalence of PTSD among the respondents was 67.5% (95% CI: 63–72). Being a merchant (AOR = 0.41 [95% CI: 0.02–0.85]), witnessing the destruction of property (AOR = 1.67 [95% CI: 1.01–2.74]), facing trauma during displacement (AOR = 6.00 [95% CI: 2.75–13.10]), frequency of displacement (AOR = 0.31 [95% CI: 0.11–0.85]), being distressed (AOR = 5.42 [95% CI: 3.25–9.05]), and unemployment (AOR = 2.09 [95% CI: 1.24–3.54]) were factors significantly associated with PTSD.

**Conclusion:**

This study provides evidence of the high prevalence of PTSD among internally displaced people. Therefore, mental health and psychosocial support are urgently required to address the identified factors and help the displaced people against long-term avoidable suffering.

## Background

According to the United Nations, internally displaced people (IDP) are “persons or groups of persons who have been forced or obliged to flee or to leave their homes or places of habitual residence, in particular as a result of or in order to avoid the effects of armed conflict, situations of generalized violence, violations of human rights or natural or human-made disasters, and who have not crossed an internationally recognized state border” [1].

There are now more than 55 million internally displaced people worldwide, of whom more than 87.2% have been displaced by conflict and violence, and 12.8% were displaced by disasters [2]. According to United Nations Human Rights Commission (UNHCR), 42% of all IDP worldwide lived in Africa. Accordingly, Ethiopia had 3.2 million IDP, which is the second highest number in Africa after the Democratic Republic of the Congo, which had more than 5 million IDP in 2019 [3]. According to this report, an estimated 358,000 people have been internally displaced in Amhara region as a result of ethnic based conflict in neighboring regions and war in the northern part of Ethiopia along Amhara and Tigray Regional borders and are in urgent need of humanitarian assistance, including many children and women who have been exposed to significant stress as a result of physical and psychological trauma [4].

Internal displacement is emerging as one of the greatest challenges facing humanity. The displacement of people leads to various mental health problems such as post-traumatic stress disorder (PTSD), depression and anxiety in these groups of people [5]. Many studies have shown that PTSD is one of the most common psychiatric disorders diagnosed in displaced persons [6].

Post-traumatic stress disorder is defined in the Diagnostic and Statistical Manual of Mental Disorders 5th Edition (DSM5), as being composed of four groups of symptoms that include intrusive and recurring memories of trauma, avoidance of trauma-related stimuli, numbness, and/or negative mood or changes in perception related to trauma and changes in reactivity and arousal [7].

Post-traumatic stress disorder has many psychosocial effects on individuals and society in general. At the individual level, victims of this disorder often experience drug use and abuse, depression, anxiety, dissociation and dissociative disorders, personality disorders, psychosis, and cognitive disorders. At a societal level, the possible consequences can be separation from families, homelessness, poverty and imprisonment [8] with.

Conflict exposes displaced populations to violence and high levels of stress, causing dramatic rises in mental illness that can continue even for long time after conflict has ceased. Displacement disrupts social support structures and exposes civilian populations to high levels of stress [9, 10].

Displaced persons may face both direct consequences, such as separation from their families, trauma, loss of properties and loved ones, and gender-based violence, and indirect consequences, such as increased malnutrition, post-traumatic stress disorder and communicable diseases [11–14].

A study done in southern Ethiopia assessed post-traumatic stress disorder by using the Post-Traumatic Stress Disorder Checklist for DSM-5 (PCL-5) and associated factors among internally displaced people found the prevalence of PTSD was 58.4% [9]. Since the conflict lasts for more than a year and the magnitude of internal displacement is high in Debre Berhan, Amhara Region, Ethiopia life stress may increase and the expected level of PTSD that can be diagnosed in this population may be presumably high.

The high numbers of internally displaced people in Ethiopia and worldwide make this study indispensable. There is still a lack of research on PTSD in IDP in African countries including Ethiopia.

The study is primarily important for planning the intervention in the treatment of PTSD in IDP in the region, for advocacy of health leaders at different health tier system to bring attention and focus to PTSD and psychosocial support during public health emergency response, recovery and rehabilitation. It also helps to produce evidence to improve health services for IDP by enhancing awareness of health leaders on PTSD. The study provides information and opportunities for integration of PTSD into primary health care in Ethiopia**.** Therefore, this study was aimed to assess the magnitude of post-traumatic stress disorder and identify associated factors among internally displaced people in camps at Debre Berhan, Amhara Region, Ethiopia.

## Methods

### Study area and period

The study was conducted in Debre Berhan, Amhara Regional State, Ethiopia. Internally displaced people due to conflict and war in the northern part of Ethiopia along Amhara and Tigray Regional borders have been residing in the camps at Debre Berhan town which is 130 km far from the capital city of Ethiopia, Addis Ababa in the northeast. By the time of the study, there were three IDP camps in Debre Berhan namely Sunflower, Teacher’s College, and China with 2956, 1340, and 568 IDP respectively. Regarding health service in the town, there are one governmental and one private hospital, three governmental health centers, four health posts, and eight private clinics providing health services to the community. Debre Berhan Referral Hospital is the only governmental hospital in the town providing preventive, curative, and rehabilitative services for 3.5 million catchment population as a referral center [15]. The data were collected from December 1–30, 2021.

### Study design and population

A community-based cross sectional study design was conducted among systematically selected internally displaced persons in camps who were aged 18 years and above in Debre Berhan, Amhara Region, Ethiopia.

### Sample size determination

The sample size was calculated by using both single and double population proportion formulae for the 1st and 2nd objectives respectively. Considering 95% confidence interval, 5% marginal error, prevalence of 58.4% from previous research in southern part of Ethiopia [9], and 10% non-response rate, the sample size was 410. The sample size for the second objective was calculated by StatCalc on Epi Info version 7.1 for four associated factors (being female, depression, displaced more than one, and witnessed the murder of family). Then comparing the sample size for both objectives, the largest sample size (410) for the first objective was taken as the final.

### Sampling procedure

Systematic random sampling was used to select study participants from the register of adult IDP. Study participants were allocated to each camp proportionally based on the number of adult IDP registered. The interval K (i.e 12) was calculated by dividing the camp population to the sampled size and proportionally allocated. The first participant was then randomly selected and the other participants were selected every 12th interval for each camp.

### Eligibility criteria

#### Inclusion criteria

Adults aged 18 years or above living in three IDP sites in Debre Berhan, Amhara region, Ethiopia.

#### Exclusion criteria

Adults aged 18 years or above living in three IDP sites in Debre Berhan who were not available in the camps with two visits were excluded from the study.

### Study variables

#### Dependent variable

Post-traumatic stress disorder (Yes/No).

### Independent variables

#### Socio-demographic factors

Sex, age, location, religion, ethnicity, family income, employment status before and after displacement, educational level, number of siblings, and any chronic illness.

#### Socio-environmental factors

Exposure to violence, availability of supportive networks, safety and protection, violence against women and children, substance use, health care, witness death of family members or friend, and witness destruction of property.

#### Displacement experience

Number of recent displacements, duration of displacement, family separation, faced any kind of trauma during displacement, and emotional experience after displacement.

### Operational definition

#### Feeling distressed

This phrase was used in this study to describe when a respondent feels restless, very distressed, for example, very upset, nervous, sad, worried, scared, or angry after being internally displaced.

#### Post-traumatic stress disorder

A total score was computed by adding the 20 items, so that possible scores range from 0 to 80 with a 5-point Likert scale (0 = not at all, 1 = a little bit, 2 = moderately, 3 = quite a bit, 4 = extremely) with a cutoff point of for PTSD development ≥33. Reliability of the PCL-5 had been tested and Cronbach’s alpha was 0.878.

### Data collection procedure

The data collection tools were adapted from PCL-5 for assessing the 20 DSM-5 symptoms of PTSD [7] and translated into the regional working languages, Amharic and back to English by language experts. The questionnaires were administered through face-to-face interview by five trained BSc Public Health professionals who were native in the local language. The data collectors were supervised by a trained General Practitioner from Ethiopian Public Health Institute.

### Data quality assurance

Before the actual data collection, the questionnaires were pre-tested in Deberk, Northern Gonder, taking 20(5%) of the total sample size, and any necessary adjustments were made accordingly. The data collectors and supervisors were healthcare professionals who received one-day training of data collection process from the principal investigator. The data collection process was strictly supervised on daily basis to achieve its intended purpose.

### Data management and data analysis

Data were collected using a pre-tested standardized questionnaire, cleaned, entered into EpiData version 3.1, and exported to SPSS version 25.0 for analysis. Binary logistic regression model was used to select candidate variables for the occurrence of PTSD among IDP at *p* < 0.25**.** Adjusted odds ratio (AOR) with 95% confidence intervals (CI) at *p* < 0.05 was used to establish the statistical significance in multivariable logistic regression to assess the association between independent variables and the occurrence of PTSD among IDP and control for confounding factors. Multicollinearity was checked by using Variance Inflation Factor (VIF) and no problems were identified (i.e.VIF < 10). Model adequacy was checked by Hosmer & Lemeshow goodness of test (*p* > 0.05).

## Results

### Socio-demographic factors

A total of 406 internally displaced people, randomly selected adults were participated giving a response rate of 99%. The median age was 35.65 years. The mean age was 39.12 (SD + 14.63) years with a range of 76 years. Majority, 357 (87.9%), of the participants were Orthodox in religion (Table 1).

**Table 1:** Socio-demographic characteristics of study participants in Debre Berhan, Amhara Region, Ethiopia, 2022

Characteristics	Category	Frequency	Percent
Sex	Male	196	48.3
	Female	210	51.7
Age (in years)	18–24	54	13.3
	25–44	222	54.7
	45–64	97	23.9
	> 65	33	8.1
Marital status	Single	69	17.0
	Married	307	75.6
	Divorced	25	6.2
	Widowed	5	1.2
Religion	Orthodox	357	87.9
	Muslim	34	8.4
	Protestant	15	3.7
Educational status	Not went to the school	132	32.5
	Elementary	168	41.4
	High school	61	15.0
	Diploma	26	6.4
	Degree & above	19	4.7
Occupation	Farmer	233	57.4
	Civil servant	25	6.2
	Merchant	71	17.5
	Student	24	5.9
	House wife	37	9.1
	Other	16	3.9
Monthly income	0–1000	28	6.9
	1001–5000	135	33.3
	5001–10,000	131	32.3
	> 10,000	112	27.6
Number of children	0	84	20.7
	1–2	81	20.0
	3–4	96	23.6
	> 5	145	35.7

### Displacement history and camp related factors

Nearly two-third of the respondents 263 (64.8%) had been displaced for first time and more than half 246 (60.6%) had been less than 6 months since displaced from their residency. Among the respondents, 207 (51%) had distressed, 224 (55.2%) had separated family members, and 228 (56.2%) had children who discontinued their education (Table 2).

**Table 2:** Displacement history and camp related factors among IDP in Debre Berhan, Ethiopia, 2022

IDP camp related factors	Category	Frequency	Percent
Frequency of displacement	Once	263	64.8
	Twice	94	23.2
	More than two times	49	12.1
Duration since displacement	< 6 months	246	60.6
	> 6 months	160	39.4
Started new work since displacement	Yes	44	10.8
	No	362	89.2
Problem of income	Yes	289	71.2
	No	117	28.8
Problem of food	Yes	153	37.7
	No	253	62.3
Problem of drinking water	Yes	71	17.5
	No	335	82.5
Problem of toilet	Yes	83	20.4
	No	323	79.6
Faced sexual violence	Yes	6	1.5
	No	400	98.5
Lacks protection from violence for women	Yes	15	3.7
	No	391	96.3

### Prevalence of post-traumatic stress disorder

The overall prevalence of PTSD among internally displaced population in the study area was 274 (67.5%) (95% CI: 63–72). Among the participants with PTSD were 130 men (47.4%) and 144 women (52.6%). Among 406 participants, those aged 45–64 years were the most affected age group, 71.1% (Table 3).

**Table 3:** Age specific distribution of post-traumatic stress disorder among IDP in Debre Berhan, Ethiopia, 2022

Age category	PTSD category		
	Yes	No	Age-specific prevalence
18–24	36	18	66.7%
25–44	148	74	66.7%
45–64	69	28	71.1%
> = 65	21	12	63.6%
Total	274	132	67.5%

### Post-traumatic stress disorder and service availability

The prevalence of PTSD among farmers and merchants were 147 (53%) and 58 (21%) respectively. Of 274 participants who developed PTSD, majority 227 (83%) had no problem of health care service, 56 (20%) had chronic illness, and 174 (64) were feeling distressed. From the study participants, 328 (80.8%) want to return back to their original residential area. The prevalence of PTSD among those participants who want to return back was 218 (66.5%). However, among 78 participants who didn’t want to return back 56 (72%) had developed PTSD. Participants who didn’t want to return back and developed PTSD mentioned fear of security, fear of host community and destruction of their property as the main challenges for not returning back.

Among 274 participants who developed PTSD, 174 (64%) were displaced once and 44 (16%) were displaced more than twice respectively. More than three-fourth of the respondents who developed PTSD, 213 (78%) witnessed destruction of their or other’s properties, 145 (53%) witnessed of death their family members or friends, whereas, 82 (30%) faced trauma during displacement.

### Factors associated with post-traumatic stress disorder

Bivariate binary logistic regression analysis was run on binary logistic regression for each independent variable with PTSD. Variables with *p*-value < 0.25 were selected as candidates for multivariable binary logistic regression analysis using enter method. Accordingly, having chronic illness, separation of family member, witnessing destruction of property, facing trauma during displacement, occupation, and monthly income were among the selected variables (Table 4).

**Table 4:** Bivariate analysis for post-traumatic stress disorder among IDP in Debre Berhan, Ethiopia, 2022

Variables	Category	PTSD		COR (95% C.I)	***P***-value
		Yes	No		
Had chronic illness	Yes	56 (20)	15 (11)	2.00 (1.09–3.70)	0.026^*^
	No	218 (80)	117 (89)	1	1
Had health care problem	Yes	47 (17)	12 (9)	2.07 (1.06–4.05)	0.034^*^
	No	227 (83)	120 (91)	1	1
Feel distressed	Yes	174 (64)	33 (25)	5.22 (3.28–8.31)	0.000^*^
	No	100 (36)	99 (75)	1	1
Separated from family members	Yes	166 (61)	58 (44)	1.96 (1.29–2.99)	0.002^*^
	No	108 (39)	74 (56)	1	1
Unemployment	Yes	177 (65)	100 (76)	1.71 (1.07–2.74)	0.024^*^
	No	97 (35)	32 (24)	1	1
Witnessed death of family or friend	Yes	145 (53)	53 (40)	1.68 (1.10–2.55)	0.016^*^
	No	129 (47)	79 (60)	1	1
Witnessed destruction of property	Yes	213 (78)	81 (61)	2.20 (1.40–3.45)	0.001^*^
	No	61 (22)	51 (39)	1	1
Faced trauma during displacement	Yes	82 (30)	8 (6)	6.62 (3.10–14.16)	0.000^*^
	No	192 (70)	124 (94)	1	1
Frequency of displacement	Once	174 (64)	89 (67)	1	1
	Twice	56 (20)	38 (29)	4.50 (1.72–11.75)	0.002^*^
	> Three	44 (16)	5 (4)	5.97 (2.17–16.44)	0.001^*^
Occupation	Farmer	147 (53)	86 (65)	1	1
	Civil servant	18 (7)	7 (5)	0.66 (0.27–1.66)	0.380
	Merchant	58 (21)	13 (10)	0.38 (0.20–0.74)	0.004^*^
	Student	16 (6)	8 (6)	0.85 (0.35–2.08)	0.729
	Housewife	28 (10)	9 (7)	0.55(0.25–1.22)	0.141
	Other	7 (3)	9 (7)	2.20 (0.79–6.11)	0.131
Income category	0–1000	13 (5)	15 (11)	2.44 (1.05–5.65)	0.038^*^
	1001–5000	98 (36)	37 (28)	0.80 (0.46–1.38)	0.417
	5001–10,000	87 (32)	44 (33)	1.07 (0.62–1.83)	0.811
	> 10,000	76 (28)	36 (27)	1	1

^*^indicates *p*-value < 0.25

In multivariable binary logistic regression analysis, those variables with *p*-value < 0.05 and 95% CI were considered as significant. Accordingly, witnessing destruction of property, facing trauma during displacement, frequency of displacement, and occupation were associated factors (Table 5).

**Table 5:** Multivariate analysis for predictors of post-traumatic stress disorder among IDP in Debre Berhan, Ethiopia, 2022

Variable	Category	PTSD		COR (95% C.I)	AOR (95% C.I)	***P***-value
		Yes (%)	No (%)			
Witnessed destruction of property	Yes	213 (78)	81 (61)	2.20 (1.40–3.45)	1.67 (1.01–2.74)	0.044*
	No	61 (22)	51 (39)	1	1	
Faced trauma during displacement	Yes	82 (30)	8 (6)	6.62 (3.10–14.16)	6.00 (2.75–13.10)	0.000*
	No	192 (70)	124 (94)	1	1	
Frequency of displacement	Once	174 (64)	89 (67)	1	1	
	Twice	56 (20)	38 (29)	4.50 (1.72–11.75)	1.49 (0.88–2.50)	0.136
	Three & more	44 (16)	5 (4)	5.97 (2.17–16.44)	0.31 (0.11–0.85)	0.023*
Monthly income^a^ (in Ethiopian Birr)	0–1000	13 (5)	15 (11)	2.44 (1.05–5.65)	1.76 (0.69–4.47)	0.234
	1001–5000	98 (36)	37 (28)	0.80 (0.46–1.38)	0.85 (0.47–1.53)	0.593
	5001–10,000	87 (32)	44 (33)	1.07 (0.62–1.83)	1.11 (0.63–1.95)	0.726
	> 10,000	76 (28)	36 (27)	1	1	
Occupation	Farmer	147 (53)	86 (65)	1	1	
	Civil servant	18 (7)	7 (5)	0.66 (0.27–1.66)	0.66 (0.24–1.79)	0.409
	Merchant	58 (21)	13 (10)	0.38 (0.20–0.74)	0.41 (0.02–0.85)	0.016*
	Student	16 (6)	8 (6)	0.85 (0.35–2.08)	0.78 (0.30–2.03)	0.612
	Housewife	28 (10)	9 (7)	0.55(0.25–1.22)	0.47 (0.19–1.14)	0.096
	Other	7 (3)	9 (7)	2.20 (0.79–6.11)	2.54 (0.78–8.30)	0.123
Chronic illness	Yes	56 (20)	15 (11)	2.00 (1.09–3.70)	1.54 (0.75–3.18)	0.240
	No	218 (80)	117 (89)	1	1	
Problem of health care	Yes	47 (17)	12 (9)	2.07 (1.06–4.05)	1.05 (0.48–2.31)	0.896
	No	227 (83)	120 (91)	1	1	
Feeling distressed	Yes	174 (64)	33 (25)	5.22 (3.28–8.31)	5.42 (3.25–9.05)	0.000*
	No	100 (36)	99 (75)	1		
Separated from family members	Yes	166 (61)	58 (44)	1.96 (1.29–2.99)	1.24 (0.75–2.04)	0.409
	No	108 (39)	74 (56)	1	1	
Unemployment	Yes	177 (65)	100 (76)	1.71 (1.07–2.74)	2.09 (1.24–3.54)	0.006*
	No	97 (35)	32 (24)	1	1	
Witnessed death of family or friend	Yes	145 (53)	53 (40)	1.68 (1.10–2.55)	1.26 (0.78–2.04)	0.345
	No	129 (47)	79 (60)	1	1	

*Indicates *p*-value < 0.05 (significant association)

^a^Indicates 1Ethiopian birr = 0.0208USD

## Discussion

This study was conducted among internally displaced people in the camps at Debre Berhan, Amhara Regional State, Ethiopia to assess the prevalence and factors associated with post-traumatic stress disorder.

The study found that the prevalence of PTSD among IDP in the study area was 67.5% (95% CI: 63–72%) consistent with a study done among survivors of terrorist attacks in Nigeria, 65.7% [16], however, it is higher than other cross-sectional studies conducted in developing countries, including Ethiopia, Uganda, Nigeria, and Darfur which report a prevalence of 58.4% [9], 54% [17], 42.2% [18], and 54% [19] respectively. This could be due to the impact of conflict, mass displacement, and the severity of property destruction experienced by these respondents, including a combination of war, political, and ethnic conflict. Several factors, such as different methodological approaches and socio-cultural factors, may also contribute to the differences in PTSD rates reported in other studies.

In this study, the overall prevalence of PTSD among the IDP was 67.5%. Of these, more than half (52.6%) were female. The result of this study is almost similar to other studies that showed that women are more likely to have post-traumatic disorders than men [17, 20]. It has been previously suggested that women may be at higher risk of mental distress because of the psychological consequences of rape, the violent loss of partner and children, and of becoming a single parent or widow.

Merchants have 59% less likely developed PTSD as than farmers (AOR = 0.41 [95% CI: 0.02–0.85]). This finding is inconsistent with a study conducted in northern Uganda after a conflict, which showed that occupation, educational level, and marital status were not associated with PTSD [21]. Similarly, the study of IDP from Iraq IDP found that socio-demographic variables were not risk factors for developing PTSD, with the exception that widowhood was a highly significant predictor [22]. This difference may be because most of the participants in the present study were farmers who lost their homes and the livestock they used to live on, rather than merchants.

Those IDP who witnessed the destruction of property were 1.67 times more likely had PTSD as compared to those who haven’t witnessed it (AOR = 1.67 [95% CI: 1.01–2.74]). The finding of this study is similar to other studies conducted in south Ethiopia and Iraq which showed that those who witnessed the destruction of personal property were 1.58 and 1.53 times more likely had PTSD than those who hadn’t experienced the destruction of personal property [9, 22] respectively.

Those IDP who faced any type of trauma during displacement were six times more likely had PTSD than those who did not experience trauma (AOR = 6.00 [95% CI: 2.75–13.10]). This finding is similar with a study conducted in southern Ethiopia, which found that cumulative trauma was significantly associated with PTSD [23].

Those who were internally displaced three or more times were 69% less likely had PTSD than first-time internally displaced persons (AOR = 0.31 [95% CI: 0.110.85]). This finding disagree with a study conducted at the IDP site in Mogadishu, which showed that respondents who had been displaced more than once were more likely to be at risk of PTSD than those who had been displaced once [16].

Participants feeling distressed were more than five times more likely than those without distressed to have PTSD (AOR = 5.42 [95% CI: 3.25–9.05]).This result is similar to a study conducted in southern Ethiopia, which showed that participants with depression were 2.6 times more likely to have PTSD compared to those without depression [9]. This might be because participants with depression are more likely to have had traumatic experiences than respondents without depression, which in turn might increases the probability of having PTSD.

Internally displaced people who were unemployed had PSTD twice as compared to their counterparts (AOR = 2.09 [95% CI: 1.24–3.54]). This finding is relatively similar to other studies conducted in Ethiopia, which showed that unemployed people were twice as likely to suffer from mental distress as compared to the employed people [23]. This similarity might be because most of the study participants were farmers who spent much of their time in the fields and currently have nothing to do all-day.

In this study, sex, age, and marital status had no statistically significant association with PTSD. This finding is supported by the study done in northern Uganda that showed PTSD has no association with sex and age [21], however, different from a study done in Iraq IDP that showed being a widow was a strongly significant predictor [22]. The finding of this study is different from other studies done in Ethiopia [9], Northern Uganda [17], and Mexico [24] which revealed PTSD was significantly associated with socio-demographic factors. The possible reason to explain why there were no age and sex differences associated with PTSD in the study area might be that the impact of traumatic events could have been almost similar across age and sex.

## Limitation of the study

Post-traumatic stress disorder is often a chronic disorder following a traumatic event and therefore longitudinal studies are likely to produce more conclusive information than cross-sectional studies. This study did not collect information on the percentage of the respondents who had been benefited from psychosocial interventions in the study area that might affect the prevalence rate of PTSD reported in this study. The PCL-5 which is a 20 item self-report measure of the DSM-5 symptoms of Post-Traumatic Stress Disorder in the past month was used in this study. To assess and diagnose PTSD, CAPS-5 is the gold standard, however, it is a sophisticated approach.

## Conclusions

This cross-sectional study found that the prevalence of PTSD among IDP in the study area is unacceptably high. The conflict in northern Ethiopia has left the study population at great risk for post-traumatic stress disorder which was higher compared to Ethiopian conflicts elsewhere. Accordingly, being a merchant, witnessing destruction of property, facing trauma during displacement, number of times changing places of living due to war, being distressed, and unemployment were significantly associated factors with the PTSD among IDP.

Therefore, the program managers and service providers should implement effective mental health services that combine medical treatment, psychological and social welfare programs for the IDP. Efforts and interventions should be made to improve the psychological wellbeing of the internally displaced population are needed to ensure preparedness for the restoration of their occupation and property when the situation improves. The Ministry of Health, Ethiopian Public Health Institute, and Regional Health Bureau should continuously intervene and assess the trends of PTSD in the displaced communities.

## Data Availability

All data generated or analysed during this study are included in this published article.

## Abbreviations

AOR: Adjusted Odds Ratio
CI: Confidence Interval
DSM-5: Diagnostic and Statistical Manual of Mental Disorders 5th Edition
IDP: Internally Displaced People
PTSD: Post-Traumatic Stress Disorder
SPSS: Statistical Package for Social Sciences
UNHCR: United Nations Human Rights Commission
VIF: Variance Inflation Factor
